# A Standards based Ontological Approach to Information Handling for use by Organizations Providing Human Tissue for Research

**Published:** 2008-04-10

**Authors:** Mary E. Edgerton, Carl Morrison, Virginia A. LiVolsi, Christopher A. Moskaluk, Stephen J. Qualman, M. Kay Washington, William E. Grizzle

**Affiliations:** 1 Department of Pathology and Laboratory Medicine, University of Texas, M.D. Anderson Cancer Center, Houston, TX; 2 Department of Pathology, The Ohio State University Hospitals, Columbus, OH; 3 Department of Pathology and Laboratory Medicine, University of Pennsylvania, Philadelphia, PA; 4 Department of Pathology, University of Virginia Health System, Charlottesville, VA; 5 Department of Laboratory Medicine, Columbus Children’s Hospital, Columbus, OH; 6 Department of Pathology, Vanderbilt University Medical Center, Nashville, TN; 7 Department of Pathology, University of Alabama at Birmingham, Birmingham, AL

## Abstract

Tissue resources have become an important component of the infrastructure of institutions as well as companies performing biomedical research. Such tissue resources may be in the model of a bank, collecting a limited type of tissues and processing and storing them following a specific protocol. Such banks or archives may be associated with a clinical study or may function indepedently. An alternative type of tissue resource is utilized by many institutions and cancer centers. In this model, the investigator specifies the methods by which selected tissues are to be collected, processed and stored. In such a “prospective model”, initially developed at the University of Alabama at Birmingham and the Ohio State University in the late 1970’s and adopted by the Cooperative Human Tissue Network in 1986, specific types of tissues are not collected unless requested by an investigator. At some sites, both a prospective and an archival (bank) model are followed. This article describes an informatics approach needed to support a prospective tissue resource. It is by necessity more complicated than a model which supports a tissue bank but also can be used by a tissue bank. Of great importance is the approach to vocabulary and common data elements needed to support the informatics system of a prospective tissue resource, especially if the informatics system is to be used by a variety of personnel with greatly varying educational backgrounds.

## Introduction

Access to human tissue for research is critical to the success of molecular medicine. The Cooperative Human Tissue Network (CHTN) is the only specialized human tissue resource funded by the National Cancer Institute^1^,^2^ that focuses on collecting tissue specimens to meet specific requirements, standards and requests of investigators. The other specimen resources listed by the National Cancer Institute are banks of archived specimens, while the CHTN is unique in that it is designed for prospective collection. Prospective collection allows the user to constrain the pre-analytical variables that can affect their research results, e.g. exclude radiation therapy in the case of a tissue donor with breast cancer.

The CHTN is comprised of six academic institutions whose mission is to provide remnant human solid tissue, fluids, and exfoliates to support biomedical researchers throughout the United States and Canada. The CHTN provides normal, malignant, benign, and diseased tissue from routine surgical resections, autopsies, and a variety of procedures resulting from the collection of body fluids. Tissues are collected prospectively; and thus as per the investigator’s protocol for collection and for preparation. Specimens are typically provided to the researcher as fresh or frozen material, fixed tissue embedded in paraffin blocks, paraffin sections of tissue mounted on microscopic slides, or as thick paraffin or frozen sections of tissues in cryotubes for molecular studies.

Currently, commercial, institutional, and academic researchers submit applications with information detailing their tissue requests and collection protocols, funding support, project objectives, and any associated institutional approvals required for working with human tissues to the CHTN division that services their geographical region. This division becomes known as the primary division for that investigator. Once the application is approved, prospective collection begins. If tissues cannot be procured for the investigator rapidly by the primary division, specifically within a reasonable amount of time, typically two weeks, then the request is networked to the other divisions. Thus, although investigators are assigned to one division, they have access to tissue from across the network as needed to fulfill their requirements. Sharing, updating and managing investigator requests for tissues among the six divisions of the CHTN requires a shared, distributed information system and sophisticated informatics support. In the past, the CHTN has relied upon stand-alone databases at each institution, with a central server for uploading networked requests from each division, merging these and distributing the merged requests to the individual divisions. With the advent of the world wide web and secure communications strategies, the CHTN has elected to migrate to a centralized relational database with web access for its tissue request and networking system. Given the complex security issues involved with utilizing a centralized infrastructure to store information protected by the Health Insurance Portability and Accountability Act of 1996 (HIPAA), the individual tissue inventories with donor information are maintained locally by each division and are not distributed unless identifying information is removed (information and reports are de-identified). Fortunately, only investigator information needs to be shared among divisions and no donor information is shared.

Any clinical annotation, such as survival times or other follow-up information, is managed by the local division. The CHTN provides diagnostic information about the tissue as a part of its tissue service. Additional information can be requested as a chart review. Chart reviews with follow-up information requested after the tissue has been distributed must follow the guidelines of the local Institutional Review Board, such as requiring informed consent by the patient for continued abstraction of their clinical record.

A database supporting a prospective tissue resource includes components related to details on the investigators, investigator requests for tissues, donor characteristics, specimen information (e.g. quality control), storage information on the tissue aliquots, integrity of storage conditions such as freezer temperature logs and information about freezer back-up systems, and the details of transfer of tissues/information to the investigator. These databases are subject to data integrity recommendations as issued by the National Cancer Institute Best Practices for Biospecimen Resources.^3^ These data integrity recommendations include guidelines for back-up systems.

Thus, the informatics tools for the CHTN include a resource to enter detailed tissue requests, as well as to retrieve investigator information about tissue requests in order to drive tissue collection. Also the system must be able to network (share) tissue requests, and to exchange information with a tissue inventory system containing donor information. The informatics requirements for this prospective collection model extend beyond the specifications of the informatics system used for an archival tissue bank. Archival tissue banks are typically designed to collect tissue using a limited number of standard collection protocols, such as snap freezing tissue in OCT using liquid nitrogen or the creation of paraffin embedded formalin fixed tissue blocks. Searching tools are important for the inventory, from which tissue collections will be withdrawn post-hoc, based on a sufficient number of tissues matching the search criteria developed by an investigator. As alluded to earlier, the business model of a prospective tissue collection also requires searching tools for the tissue request system in order to anticipate and plan for specific needs of future collections.

The inherent design of each component of such a database is different; thus, a tissue request database is different from a tissue collection/storage database; however, the data elements and allowable values for all data elements that are common to the components of the database must be consistent to permit effective communication. Similarly, if different organizations want to have their databases to communicate, standardization of data elements (common data elements) across organizations would be critical to effective communication.

Recently, the National Cancer Institute, in recognition of a need for common data elements for computer systems so that cancer centers and other groups of investigators can exchange large volumes of data, instituted the Cancer Bioinformatics Grid (CaBIG) projects to provide standards for data exchange in cancer research.^4^–^10^ They also were to develop and provide a variety of generic software systems and tools to assist in compliance with caBIG standards. One of the tools currently offered is caTissue, a tissue inventory for archived tissue collections. The informatics requirements for the CHTN, which centers upon the prospective tissue collection model, extend beyond those for caTissue. As a result, the CHTN has constructed a complementary informatics system called TissueQuest© for managing tissue requests within the context of its business model for prospective collection and rapid distribution of tissues.

One of the business requirements for TissueQuest© was that it be accessible to a spectrum of users with a wide variety of educational backgrounds. The user population includes pathologists who oversee and provide quality control for the tissue collections, division coordinators who manage repository operations and tissue procurement personnel who collect and store the tissue and distribute tissues to researchers. In addition, information concerning tissue requests that is compatible with the system must be obtained from donor records and shared with research investigators, ranging from basic scientists to physician investigators, as well as their laboratory managers who may not have advanced degrees. A simplified ontological approach to the tissue request system appropriate for several levels of sophistication has been developed for this application such that it meets the critical requirement that the vocabulary be useful over various levels of experience and education. The data model incorporating the ontology has been constructed using concepts based on anatomy, the etiology and the pathophysiology of the disease. In addition to meeting its own user requirements, the CHTN has worked to remain compliant with the complementary efforts of caBIG to standardize data elements for tissue collection and annotation for neoplastic tissues.

A particular conundrum has been the selection of a terminology source for annotation of the tissue. There are two perspectives on annotation of tissue. One is that it should be based upon an accepted standard in disease terminology, such as the International Classification of Diseases (ICD) system, or its counterpart for cancer, the Oncology Classification of Diseases (OCD). The other is that it should be based upon an accepted standard in the terminology of histopathologic diagnoses, such as the College of American Pathologists (CAP) cancer protocols or the World Health Organization (WHO) histological classification of tumors. The CHTN has adopted the latter approach, which uses histological diagnoses as a defining property of tissue, while disease terminology is considered a property of the patient. This is important in that many tissue resources have primary access to the surgical pathology report which may or may not include information on systemic disease processes (e.g. specifically for an invasive tumor from a patient with diabetes; thus, the tissue resource is unlikely to have information about the patient’s systemic disease). The CHTN has further extended this concept such that both the tissue request and the tissue annotation are based on the histological diagnosis. While tissue can be requested from patients who have certain characteristics, the tissue itself is parent to its histological properties and pathophysiological characterization. Non-histological disease descriptors can be used to qualify a histological annotation, such as selecting a grade or stage of disease, or to pre-define the donor population, such as patients with breast cancer who have a history of ovarian carcinoma.

Having adopted the concept of a histological diagnosis as the focal point for ***both*** the tissue request and the tissue annotation, it is still necessary to select a terminology that will become the allowable values for the common data elements each corresponding to a specific histological diagnosis. This issue is complicated because diagnostic pathology has suffered from a lack of standards for terminology in assigning a diagnosis. For example, squamous cell carcinomas of the lung have also been called epidermoid carcinomas of the lung. This synonymous terminology is a source of difficulties for investigators who use tissues in research and bio-specimen repository personnel who are not trained in pathology. The problem of choosing a terminology is complicated further by the fact that there is not always a one-to-one mapping of histological disease definitions between terminology sources, including the CAP and the WHO, a problem that is compounded by the evolution of disease organization and terminology over time.

This paper describes both the disease classification data model developed for TissueQuest© and the approach used in selecting the terminology for histological diagnoses. A complete list of allowable diagnostic terms for all tissue request categories is available in Appendix A which is posted on the website.

## CHTN Disease Classification Data Model for TissueQuest©

The ontology for the new disease classification system of the CHTN organizes tissue requests at a superficial level using a combination based on the pathophysiology of the disease, the metadata required for making a tissue request, and the types of requests commonly encountered by the CHTN. The pathologists associated with the CHTN provide the knowledge basis for the pathophysiology of disease; however, the knowledge basis for the metadata required for making tissue requests and for understanding the types of requests commonly encountered results from the over twenty years of experience across the network in receiving requests for tissues and supplying researchers with appropriate tissues. Our experience can be considered as the “human factor,” which is critical to the success of any informatics system. It insures that the system will operate efficiently within the context of the flow of information that normally occurs between an investigator or designate and the personnel of the tissue resource as they assist the investigator in navigating the domain of diagnostic pathology with a scientific destination in mind. It encompasses knowledge gained from multiple decades of experience across the CHTN.

**Figure 1:**
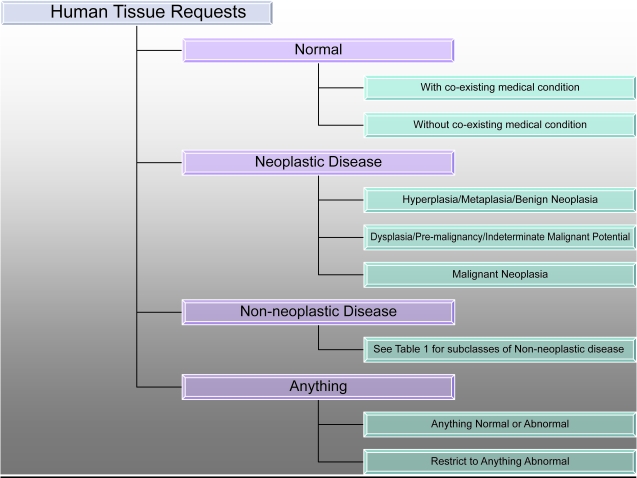
CHTN high level ontology for TissueQuest©.

As depicted in Figure 1, there are four broad classifications for tissue requests: Normal Tissue not related to the patient’s diseases, Tissue from patients with a Non-Neoplastic Disease, Tissue, diseased and non-diseased, from patients with a Neoplastic Disease, and Anything (or Any), which is any of these major categories. The role of pathophysiology in the data model is clear for the normal versus diseased categories. The inclusion of a category for “Any” or “Anything” is derived from the CHTN experience, or the human factor.

As Figure 1 also shows, requests for “Normal Tissue” are further subdivided according to whether or not co-existing medical conditions are acceptable or not, while the Anything category is further subdivided into Anything Normal or Abnormal and Anything Abnormal. The Neoplastic Disease Category is further subdivided into the following three categories: Benign/Hyperplasia/Metaplasia, Dysplasia or Neoplasia of Indeterminate (Borderline) Malignancy, which includes tumors such as intra-adrenal paragangliomas and borderline tumors of the ovary, and frankly Malignant tissues. The Non-neoplastic Disease Categories are the most numerous and are not depicted in Figure 1. These are listed in Table 1. Non-neoplastic disease is broken down further by etiology inasmuch as it is understood and by organ site.

The business rules of the CHTN are such that all types of tissue requested by a single investigator are included within a single investigator file and within this file, a single request is made up of all required requests related to a single organ. For example, if the investigator requests ductal carcinoma of the breast and requires matching normal breast tissue, as well as a 0.2 ml of serum from the same donor, this would be a single request. If all these tissues (e.g. serum) can come from any patient, then multiple tissue requests are generated. Thus, when generating reports, the tissue collectors know that tissues requested within a single tissue request must come from the same donor and that any number of related specimens within a single tissue request must come from the same donor. Any number of related specimens or body fluid/exfoliates are allowed. This business rule also governs the metadata collected for each tissue request, which will be discussed in more detail subsequently.

Users first select a tissue request type from the high level ontology classes above: Normal, Neoplastic Disease, Non-Neoplastic Disease, and Anything. To simplify the system for the coordinators, the menu for tissue type lists the three Neoplastic Disease subclasses instead of Neoplastic Disease. Thus, coordinators do not have to click twice to reach this level.

For Normal and Anything tissue request types, the histological diagnosis has already been selected. The user then selects solid tissue and/or body fluids. The terminology for these will be discussed below. In the case of neoplastic diseases, the user defines the neoplastic disease of the donor by selecting the primary organ site and the disease. Again, by experience, a category of “Any” is available to incorporate requests such as “Malignant Tissue, Any Type”. Such requests are sometimes made, for example, to test a new antibody. Once the disease has been defined, e.g. adenocarcinoma of the colon, then the user selects the tissue types requested: primary tumor, grossly uninvolved tissue, and/or tumor interface with uninvolved tissue. The user can also select another unrelated body site for tissue, and/or a metastasis. The list of sites for a metastasis selection will be described subsequently.

## Terminology for Anatomic Sites, Sub-sites, and Body Fluids

Non-neoplastic disease is similar to neoplastic disease in that the user first selects the disease, then a site (presumed involved) for tissue collection. The user is given the option to collect matched uninvolved tissue from the same site. The anatomic sites and body fluids lists will be described in the next section.

The selection of the terminology for the tissue request types will be discussed following a description of the terminology for the anatomic site, sub-sites and body fluid.

## Terminology for Anatomic Sites, Sub-sites, and Body Fluids

### Anatomic sites and subsites

If solid tissue is selected, then the user is given a list of anatomic sites from which to choose. A single list of anatomic sites and sub-sites is used to characterize solid tissues requests for all types of tissue requests except Non-neoplastic disease requests, which will be described below. The anatomic site triggers an associated list of subsites. The list of anatomic sites and associated sub-sites is also available on the journal website as Appendix B.

The anatomic sites list is built upon the organization of anatomic sites and associated data elements in the CAP Cancer protocols, with modifications and enhancements to meet the needs of the CHTN. For example, to date researchers have never specified laterality in their requests for breast or other bilateral tissue, so this has been excluded, but could be added if necessary. An anatomic site termed vascular, not included in the CAP protocols, is given as a choice. It permits a number of sub-sites to be specified including “any” or alternatively, a specific artery or vein. For example, a portion of a renal artery frequently is requested by investigators.

The neoplastic or the non-neoplastic disease site list for tissues from an unrelated body site uses a concatenated list of anatomic sites and subsites. The decision to concatenate the two was based on a desire to optimize performance and training and eliminate triggering of subsequent drop-down lists in the software by the tissue request system. This performance factor was weighed against making lists as short as possible, and therefore easier for the end-user to use. This is in contrast to tissue requests for normal, anything, or neoplastic diseases where the histology is a function of the anatomic site. Therefore, because anatomic site was required to trigger the list of possible histologies, there is no computation time added to trigger a list of subsites.

In the case of non-neoplastic disease, the disease itself is not always defined by or localized to a single organ or tissue type. For example, infections can occur in many different sites. Also, an organ specific disease such as cardiomyopathy may have downstream effects on the liver. Thus, the decision was made to minimize the performance time required and to use the concatenated anatomic site-subsite list. For purposes of illustration, this list is given in Appendix C.

### Body fluids

In addition to solid tissues, the user can request a body fluid or exfoliate. These lists differ slightly between the normal type tissue request and the other categories because certain body fluids have a specific site from which they can be collected, e.g. a pleural or pericardial fluid. The body fluid selection list is based on CHTN requests, and uses community standard terminology for the topology of the fluid. The lists of options for Normal and the other tissue request types is included in Appendix D and on the journal website as supplemental data.

### Metastatic site selections

For metastatic sites, we created a list with terminology compliant with our list of anatomic sites and subsites, but based on actual requests to the CHTN. An important designation is whether or not only metastatic tissue is to be collected, if both primary and metastatic tissue are required, or if either primary or metastatic tissue is needed. Similarly, the site of the metastasis may be specified or alternatively “any” is used as the modifier when metastatic tissue from any site is acceptable. This is given in Appendix E and on the journal website as supplemental data.

## Terminology for Subsite Qualifier for Normal and Anything Tissue Requests

Investigators often characterize their normal tissue requests beyond the level of an anatomic site and subsite. For example, only mucosa may be requested. In addition, normal tissue under the effect of certain hormones might be requested, such as lactating breast. The list of subsite qualifiers is based upon the Normal tissue requests that the CHTN has received. As mentioned earlier, the subsite qualifiers for normal tissue requests are included in Appendix A.

### Non-neoplastic disease metadata and terminology

Non-neoplastic disease is broken down further by etiology inasmuch as it is understood and by organ site. The main groups are given in Table 1. The philosophy has been to extend the complexity of the description and narrow the focus on each disease class during future operations of the CHTN, with additional experience causing new tissue requests to be incorporated into the database. As discussed previously, future plans include the development of a concept based interface that would enable investigators to browse the selections based on some knowledge of the etiology or pathophysiology of the disease, e.g. an autoimmune disease or diseases with Mendelian inheritance patterns (e.g. pancreatic tissues from patients with cystic fibrosis).

While this list is not exhaustive, it includes all of the disease entities that have been requested to date from the CHTN. Addition of new diagnostic terms to the database can be easily added to the system as needed.

## Neoplastic Disease Terminology

### General considerations

Neoplastic disease has been subdivided into the following three main categories, based upon their expected behavior: Benign/Hyperplasia/Metaplasia, Dysplasia or Neoplasia of Indeterminate (Borderline) Malignant Potential, or Malignant (frankly so). As described earlier, a neoplasia is typically a function of its anatomic site except for lymphomas, sarcomas and a few other diagnoses. We have created this data model using the basic concept that epithelium is specialized for function within an organ or anatomic site, and thus an adenocarcinoma of the lung is inherently different from an adenocarcinoma of the colon. Therefore, disease lists are triggered by site. For example, the term “adenocarcinoma” may be found for multiple anatomic sites. Once selected in combination with an anatomic site, it represents the primary adenocarcinoma of that organ site. Users may also specify any anatomic site and request adenocarcinoma. Thus, an adenocarcinoma from any site will be procured for the investigator.

In some cases, the organ of origin is not regionally delimited with the body, such as hematopoietic tissue (e.g. lymphomas) or soft tissue (e.g. liposarcomas). Neoplasia for these can either be requested on a systemic basis, e.g. with soft tissue as the anatomic site for sarcoma, or originating in a delimited anatomic location, e.g. an angiosarcoma of the breast. While there is, in this example, a replication of the histological diagnosis term or value “angiosarcoma” in both the soft tissue and the breast disease lists, the union of the anatomic site and the disease itself combines to create unique tissue requests: “Breast-Angiosarcoma” versus “Soft Tissue-Angiosarcoma” and as such are not synonymous. Another possibility would be to restrict the angiosarcoma diagnosis to a major anatomic site category of soft tissue, but let the user select a specific site for it. While this is satisfactory from a taxonomic perspective, it would require the users to have a very high level of knowledge of anatomy, diagnostic pathology, as well as the pathophysiology of all diseases. Specifically, tissue procurement personnel would need to learn which diseases are primary to a specific organ site (carcinomas) and which diseases can be found anywhere (e.g. most sarcomas). Further, they would need to be able to recognize those sarcomas that are specific to a site, e.g. malignant phyllodes tumor that is found only in the breast or have special pathophysiologic characteristics such as angiosarcomas associated with the breast.

A decision was made to compromise here in order to ease the management of tissue collection and distribution by repository personnel who are not trained as pathologists. Thus, when inputting a tissue request for a type of sarcoma, the personnel do not need to know that the embryologic origin of this tumor is mesenchyme, all the sites where specific types of sarcomas arise, or special sarcomas that arise because of a specific condition (e.g. angiosarcoma of the breast). Even so, investigators may request a mesenchymal tumor from any soft tissue site, or may specify the site and then a tumor whose embryologic origin is mesenchymal or epithelial. In future versions of the system that include a concept based interface, users can be guided to select the appropriate disease categories via the search engine and a series of questions.

Using the same philosophy of compromise between a very granular, purely biologically defined system and a system that can be used by non-pathologists, the CHTN has combined hyperplasia and metaplasia with benign neoplasia as a subcategory of neoplastic disease, as well as dysplasia, premalignant tumors, and tumors of unknown malignant potential.

As stated earlier, the terminology for neoplastic disease is included in Appendix A. The adoption of a terminology for cancer diagnoses requires more discussion than the previous categories because of the desire to remain compliant with tissue banking tool development by caBIG.

## Terminology for Neoplasia, Standards, and CaBIG

As of January 1, 2004, the American College of Surgeons (ACS) Commission on Cancer (CoC) mandated new standards for pathology reports, including standards for histopathological terminology, through its certification program. One new standard requires that pathologists at CoC-approved cancer programs include all scientifically validated or regularly used *data elements* and *their allowable values* from the CAP Cancer Protocols checklists in their reports for each site and specimen. In order to comply with the ACS mandate, cancer programs are now expected to show that 90 percent of pathology reports include all scientifically validated or regularly used elements of the appropriate checklists.

The CAP Cancer Protocols checklists for invasive carcinomas, and where they are included, diseases of indeterminate malignancy were adopted by the CHTN as a starting basis for the neoplastic disease terminology. *This is because CHTN personnel have ready access only to the surgical pathology report that is tied to a specific tissue.* The CAP Cancer Protocols and checklists have been assembled, organized, and approved by experts for the carcinomas. For soft tissue and bone tumors, the CAP has recently issued a protocol in which they refer the user to histopathological terminology approved by the World Health Organization terminology. The CHTN has also adopted the terminology approved by the World Health Organization for bone and soft tissue tumors.

In a limited number of instances, tissues have been requested for entities that are still considered controversial. In these instances, the literature was reviewed and nomenclature was selected that fulfilled the following two criteria: it was proposed by experts, and it represented the most granular disease definition that was current.

By adopting the CAP protocols and checklists for malignant tissue requests, TissueQuest© at least includes terminology that is used by CaBIG for malignancy. The Tissue Banking and Pathology Tools Workspace of the Cancer Bioinformatics Grid effort has recently published a case study in which they demonstrated how the CAP reporting protocols for at least three cancer systems would be integrated into caCORE.^10^ They concluded that “The CAP cancer checklists can be used as the basis for an electronic data standard in pathology using the caBIG semantic modeling methodology.” In addition, adoption of the CAP cancer protocols simplifies the process of matching a tissue request to diagnostic terminology used by pathologists at institutions that are approved by the ACS. We propose that the main problem with this approach is that it ignores the educational level and lack of detailed training in pathology of those personnel (e.g. tissue resource personnel and investigators) who will be using the database.

The CAP checklists are adequate for the vast majority of frankly malignant or invasive carcinomas; however, they do not address pre-malignancies, most dysplasias, and benign tumors, nor do they consider non-neoplastic diseases (e.g. idiopathic pulmonary arterial hypertension). (Bone and soft tissue terminology is the same as that proposed by the WHO, as stated earlier). The CHTN has assembled a terminology for these lesions using the approach described above for controversial lesions: the terminology 1) is used by experts in the field and 2) it can offer sufficient granularity at the diagnosis level to define tissue, with the caveat that proper names associated with a lesion should be avoided.

## Conclusion

The CHTN was presented with a problem: the need for an informatics system for managing tissue requests that 1) would use histological definitions to annotate the tissue, 2) would be caBIG compliant within the domain of cancer tissues and diagnoses, and 3) that could be used by pathologists and and most importantly by non-pathologists. In order to structure this system, an ontology was built for disease classification, and the tissue request system structure was built upon its backbone. Within the ontology, data elements were selected to supply the appropriate metadata for each tissue request. These were selected for compliance with caBIG as far as diagnostic data elements were concerned. However, additional data elements beyond the scope of those used by caBIG tools have also been introduced as needed. Some of these have been discussed here. The tools for collecting and storing information concerning actual tissue collection protocols will be discussed in detail in a subsequent publication describing TissueQuest© itself in more detail. Finally, a set of allowable terms for diagnostic histopathology for use by the CHTN was assembled based upon the data model for disease classification. For malignant neoplasia, CAP terminology for carcinomas and WHO terminology for bone and soft tissue tumors has been adopted, supplemented as needed using terminology that has been accepted by experts in the field if the terminology is not included in CAP protocols for carcinomas or WHO protocols for bone and soft tissue tumors, but is sufficiently granular to define a histopathological diagnosis, including the caveat that entities with proper names are minimized.

This ontological structure provides a number of advantages. A search engine has been superimposed on it so that personnel can search by organ site, disease class, or disease name, and they will be returned a list of options to choose from. A particular option can be selected and will pre-fill a tissue request with the information contained within the selection. This search engine will be further enhanced with maps to other vocabularies and to NCI/CaBIG approved concepts. In addition, a concept based interface that guides users using a decision tree overlaid on the ontology developed here is planned for the future. It is anticipated that this kind of interface will make this tool amenable to investigators from a broad range of educational backgrounds.

The CHTN also plans to submit its data elements and allowable values to the Cancer Data Standards Repository (CaDSR) so that they can be used to extend the domain of tissue procurement currently defined by caTissue, an archival based tissue inventory system supported by caBIG. In addition to metadata for defining tissue procurement protocols, the ontology and terminology for diseases not included in the domain of caBIG are included here for use by the tissue collection community.

Because malignancy of the breast is relatively complicated, several examples of how requests for such tissue would be handled by our vocabulary system are described as follows:

1. An investigator requests breast cancer. The investigator is contacted to determine if a special type (e.g. ductal, lobular, tubular etc.) is requested. If the investigator wants any carcinoma, a very common request, the terminology used would be breast, invasive carcinoma, (NOS). The coordinator can either generate a Malignant tissue request and select these values via the drop-down lists for anatomic site and histologic diagnosis, or use the search engine to search the selections for a malignant breast tissue request using the keywords “malignant” and “breast”. (The search engine defaults to the Boolean operator AND to minimize the selections that it retrieves.). The coordinator selects Breast-Invasive Carcinoma (NOS), and a tissue request form is generated that has been automatically populated with these terms for the anatomic site and histologic type.
2. If the investigator specified only primary mucinous breast carcinoma tissue, the tissue request type would be malignant and the anatomic site and histologic type would be breast and mucinous. As above, these could be entered on a malignant tissue request page, or a malignant tissue request page populated with these values for anatomic site and histologic type could be generated via the search engine. The coordinator would request Primary Anatomic Site as the tissue type requested by the user.
3. If an investigator requested tubular carcinomas from African-Americans between 20 and 50 years of age, but only metastases to lymph nodes (any site) was needed, the malignant tissue request could be generated via the search engine as described above. For tissue type, the coordinator would select Metastasis and would choose lymph nodes-any as the tissue site. There is an option to specify age range, gender, and race/ethnicity on the tissue request page.

In summary, the CHTN has developed a vocabulary system with an associated search engine that is user friendly and is easily used by personnel with varying educational backgrounds and without extensive training in diagnostic pathology or a related field. This vocabulary and associated search engine is available at no cost via requests submitted to the CHTN Central coordinator: chtncentral@earthlnk.net.

## Supplementary Material

Appendix A

Appendix B

Appendix C

Appendix D

Appendix E

## Figures and Tables

**Table 1:** Major disease classes for non-neoplastic disease.

Any
Acquired Cysts
Congenital-Developmental
Diseases with Mendelian Inheritance
Ectopic
Infection
Infection-Cardiovascular
Infection-Gastrointestinal Tract
Infection-Peritoneum
Infection-Products of Conception
Infection-Products of Conception
Infection-Prostate
Infection-Pulmonary
Infection-Renal
Infection-Reproductive Tract
Infection-Upper Respiratory Tract
Infection-Vascular
Infection-Specify Site
Inflammatory-Non-Infectious
Inflammatory-Non-infectious-Allergic
Inflammatory-Non-infectious-Allergic-Skin
Inflammatory-Non-infectious-Autoimmune
Inflammatory-Non-infectious-Autoimmune-
Cardiovascular
Inflammatory-Non-infectious-Autoimmune-Endocrine
Inflammatory-Non-infectious-Autoimmune-
Hematologic
Inflammatory-Non-infectious-Autoimmune-
Hepatobiliary
Inflammatory-Non-infectious-Autoimmune-Musculo-
Skeletal
Inflammatory-Non-infectious-Autoimmune-Skin
Inflammatory-Non-infectious-Automimmune-
Hepatobiliary
Inflammatory-Non-infectious-Automimmune-
Systemic/Connective Tissue
Inflammatory-Non-infectious-Automimmune-
Systemic/Connective Tissue/Vascular
Inflammatory-Non-Infectious-Bone
Inflammatory-Non-infectious-Cardiovascular
Inflammatory-Non-infectious-Exposure-Pulmonary
Inflammatory-Non-Infectious-Gastrointestinal Tract
Inflammatory-Non-Infectious-Genitourinary Tract
Inflammatory-Non-infectious-Hepatobiliary
Inflammatory-Non-infectious-Idiopathic-Endocrine
Inflammatory-Non-infectious-Idiopathic-
Gastrointestinal Tract
Inflammatory-Non-infectious-Idiopathic-Pulmonary
Inflammatory-Non-infectious-Idiopathic-Reproductive
Tract
Inflammatory-Non-infectious-Idiopathic-Systemic
Inflammatory-Non-infectious-Musculo-Skeletal
Inflammatory-Non-infectious-Reproductive Tract
Inflammatory-Non-infectious-Salivary Gland
Inflammatory-Non-infectious-Skin
Inflammatory-Not Specified by Etiology
Inflammatory-Not Specified by Etiology-
Cardiovascular
Inflammatory-Not Specified by Etiology-Connective
Tissue
Inflammatory-Not Specified by Etiology-Endocrine
Inflammatory-Not Specified by Etiology-
Gastrointestinal Tract
Inflammatory-Not Specified by Etiology-Genitourinary
Tract
Inflammatory-Not Specified by Etiology-Musculo-
Skeletal
Inflammatory-Not Specified by Etiology-
Oropharyngeal
Inflammatory-Not Specified by Etiology-Reproductive
Tract
Inflammatory-Not Specified by Etiology-Respiratory
Tract
Inflammatory-Not Specified by Etiology-Salivary
Glands
Liths
Medical Kidney
Neurodegenerative/Neuropsychiatric
Non-inflammatory Diseases/Lesions
Non-inflammatory Diseases/Lesions-Cardiovascular
Non-inflammatory Diseases/Lesions-Gastrointestinal
Non-inflammatory Diseases/Lesions-Genitourinary
Non-inflammatory Diseases/Lesions-Hepatobiliary
Non-inflammatory Diseases/Lesions-Metabolic
Non-inflammatory Diseases/Lesions-Metabolic-
Cardiovascular
Non-inflammatory Diseases/Lesions-Metabolic-
Musculo-Skeletal
Non-inflammatory Diseases/Lesions-Musculo-
Skeletal
Non-inflammatory Diseases/Lesions-Ophthalmic
Non-inflammatory Diseases/Lesions-Pulmonary
Non-inflammatory Diseases/Lesions-Reproductive Tract
Non-inflammatory Diseases/Lesions-Skin
Non-inflammatory Diseases-Idiopathic-Hematologic
Paraneoplastic Syndromes
Structural-Ectatic-Etiology Not Specified
Structural-Etiology Not Specified
Structural-Mechanical-Etiology Not Specified
Syndromes not well categorized
Transplant Related
Trauma
